# Comprehensive Genomic Analysis of *Pseudomonas aeruginosa* PSU9449 Isolated from a Clinical Case in Thailand

**DOI:** 10.3390/antibiotics14060530

**Published:** 2025-05-22

**Authors:** Thitaporn Dechathai, Kamonnut Singkhamanan, Thunchanok Yaikhan, Sarunyou Chusri, Rattanaruji Pomwised, Monwadee Wonglapsuwan, Komwit Surachat

**Affiliations:** 1 Department of Biomedical Sciences and Biomedical Engineering, Faculty of Medicine, Prince of Songkla University, Songkhla 90110, Thailand; oumthitaporn2543@gmail.com (T.D.); skamonnu@medicine.psu.ac.th (K.S.); ythuncha@medicine.psu.ac.th (T.Y.); 2 Division of Infectious Diseases, Department of Internal Medicine, Faculty of Medicine, Prince of Songkla University, Songkhla 90110, Thailand; sarunyouchusri@hotmail.com; 3 Division of Biological Science, Faculty of Science, Prince of Songkla University, Songkhla 90110, Thailand; rattanaruji.p@psu.ac.th (R.P.); monwadee.wo@psu.ac.th (M.W.); 4 Translational Medicine Research Center, Faculty of Medicine, Prince of Songkla University, Songkhla 90110, Thailand

**Keywords:** *Pseudomonas aeruginosa*, multidrug-resistant bacteria, whole-genome sequencing (WGS), bioinformatics analysis, mobile genetic elements (MGEs)

## Abstract

**Background/Objectives:** *Pseudomonas aeruginosa* is one of the most significant multidrug-resistant bacteria. It poses considerable challenges in terms of treatment and causes hospital-acquired infections that lead to high morbidity and mortality. Colonization by *P. aeruginosa* in a patient without clinical signs of infection is a concern in hospital settings, as it is an opportunistic pathogen and can potentially be a multidrug-resistant strain. The objective of this study was to characterize and provide a detailed genomic analysis of this strain of the *P. aeruginosa* PSU9449 genome, an isolate obtained from a patient at Songklanagarind Hospital, Thailand. **Methods**: Whole-genome sequencing (WGS) and bioinformatics analysis were employed to examine the genomic features of *P. aeruginosa* PSU9449. We performed sequence type (ST) determination through multilocus sequence typing (MLST), identified antimicrobial resistance genes (ARGs), virulence factor genes (VFGs), and analyzed the presence of mobile genetic elements (MGEs). Additionally, we compared the PSU9449 genome with strains from neighboring countries to understand its phylogenetic relationship. **Results**: The *P. aeruginosa* PSU9449 genome contained five insertion sequences and several ARGs, including *fosA*, *aph (3’)-IIb*, *bla*_OXA-50_, and *catB7*. It also harbored VFGs related to flagella (*fli*, *fle*, and *flg*), the type 6 secretion system (*hcpA*, *tssA*, and *las*), and the type 3 secretion system (*exoS*, *exoU*, and *exoT*). MLST identified PSU9449 as ST3777, which was reported in Thailand for the first time. Phylogenetic analysis based on core gene SNPs revealed that PSU9449 was closely related to *P. aeruginosa* HW001G from Malaysia and *P. aeruginosa* MyJU45 from Myanmar, forming a distinct clade. **Conclusions**: This study presents a comprehensive genomic analysis of *P. aeruginosa* PSU9449, shedding light on its genetic characteristics, antimicrobial resistance profile, and virulence potential. Interestingly, ST3777, the novel STs from the published genomes of *P. aeruginosa* in Thailand, were assigned in this study. The findings enhance valuable insights into the expanding knowledge of *P. aeruginosa* PSU9449 and highlight the importance of ongoing surveillance of its genetic diversity.

## 1. Introduction

*Pseudomonas aeruginosa* is a Gram-negative rod bacterium and an opportunistic pathogen classified as one of the ESKAPE pathogens (*Enterococcus faecium*, *Staphylococcus aureus*, *Klebsiella pneumoniae*, *Acinetobacter baumannii*, *Pseudomonas aeruginosa* and *Enterobacter* species), a group of multidrug-resistant (MDR) bacteria that play a major role in outbreaks of nosocomial infections worldwide [1]. The MDR-ESKAPE pathogens in Southeast Asian countries are becoming increasingly prevalent [2,3,4]. These pathogens are notorious for their resistance to multiple antibiotics, complicating treatment and leading to prolonged ICU stays, higher healthcare costs, and increased mortality rates [5]. MDR *P. aeruginosa* was highlighted in the CDC’s 2019 Antibiotic Resistance Threats Report, which noted that in 2017, there were approximately 32,600 hospital-acquired infections caused by MDR *P. aeruginosa* and approximately 2700 associated deaths in the United States [6]. MDR *P. aeruginosa* is a significant global health concern and has high prevalence in various regions, particularly in Africa [7,8]. There is high-level resistance to carbapenems, cephalosporins, aminoglycosides, and colistin in some areas. Additionally, MDR *P. aeruginosa* has a high incidence in Europe and the Asia-Pacific region [9,10,11].

Currently, whole-genome sequencing (WGS) has become popular in several research areas. WGS is a powerful tool for studying comprehensive genetic information, particularly for identifying antimicrobial resistance genes (ARGs), mobile genetic elements (MGEs) and virulence factor genes (VFGs). In 2023, a study provided genomic insights into colonizing isolates of *P. aeruginosa* in Southern Thailand using WGS. The findings revealed frequently acquired ARGs such as *fosA*, *aph (3’)-IIb*, and *bla*_OXA-50_, which confer resistance against beta-lactams, aminoglycosides, and fosfomycin. Furthermore, a novel sequence type, ST-3910, was identified for the first time, proving useful for monitoring *P. aeruginosa* outbreaks across several regions [12]. Despite these advances, a significant knowledge gap remains in understanding the genomic characteristics and regional diversity of MDR *P. aeruginosa* isolates, particularly in Southeast Asia. While several global studies have provided a broad perspective on MDR strains, comparative genomic analyses at a local level between Thailand and neighboring countries remain limited. Such comparative studies are crucial for identifying unique genetic traits, resistance mechanisms and transmission that may be influenced by regional healthcare practices, environmental factors, or antibiotic usage patterns.

Given the global significance of MDR *P. aeruginosa* and its impact on public health, this study aimed to characterize the genomic profile of the strain PSU9449, isolated from a patient at Songklanagarind Hospital, Thailand. Using WGS and bioinformatics analysis, we identified ARGs, MGEs, and VFGs. Additionally, a comparative genomic analysis was conducted to examine genetic similarities and variations between PSU9449 and strains from Cambodia, Malaysia, and Myanmar, providing insights into regional genomic diversity. We hypothesize that MDR *P. aeruginosa* PSU9449 harbors distinct antimicrobial resistance determinants, mobile genetic elements, and virulence factors that contribute to its clinical significance. Furthermore, we expect that comparative genomic analysis will reveal regional variations in genetic composition, influenced by local antibiotic usage patterns, environmental factors, and healthcare practices. These findings will provide critical insights for managing infections and preventing the spread of resistant strains.

## 2. Results and Discussion

### 2.1. Clinical Information and Antimicrobial Susceptibility Profiles

*Pseudomonas aeruginosa* PSU9449 was isolated from the throat swab of a patient in the male medicine ward. The patient was admitted with underlying diseases but without clinical signs of a *P. aeruginosa* infection. This isolated species was identified as *P. aeruginosa,* according to routine laboratory procedures. The antimicrobial susceptibility results indicated that the strain was sensitive to all tested antibiotics.

### 2.2. Genome Characteristics and Species Confirmation

**Figure 1:**
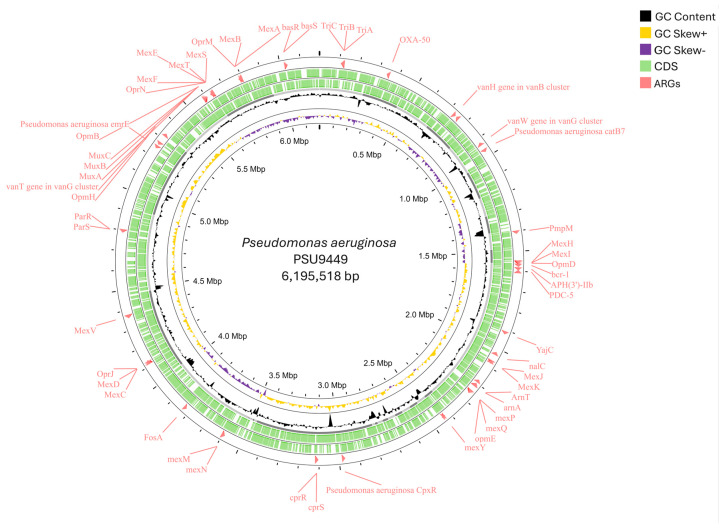
Circular draft genome mapping of the *Pseudomonas aeruginosa* PSU9449 (accession number JAWPGW000000000) revealed a genome size of 6,195,518 bp. The CGView tool visualized the circular genome, which contained genomic features represented from the outer to the inner circle. Circle 1 represents the antimicrobial resistance genes (ARGs) while circles 2,3 represent the forward and reverse strands of CDS, respectively. The innermost circles represent the GC content, GC skew+, and GC skew−.

The whole-genome sequencing provided 37 contigs. The genome assembly size of *P. aeruginosa* PSU9449 was 6,195,518 bp with a GC content of 66.4%. The N50 of the genome was 399,445 bp, while the L50 was contig 6. Genome annotation revealed a total of 5683 genes, including 5607 coding sequences (CDS), 68 tRNAs, four rRNAs, one tmRNA, and three CRISPRs. General information, including 51 predicted antimicrobial resistance genes (ARGs) and 284 predicted virulence factor genes (VFGs), based on bioinformatic analysis of the draft genome assembly, is provided in Table 1. A circular map representing the draft genome and the predicted locations of resistance genes is shown in Figure 1.

To confirm the species, the ANI criterion, most often using values >95%, was compared with the reference strain. In this study, *P. aeruginosa* PSU9449 exhibited 99.28% ANI to the reference strain *P. aeruginosa* PAO1. The predicted serotype of *P. aeruginosa* PSU9449 was O11. This result was consistent with previous studies reporting that the most prevalent serotypes of *P. aeruginosa* were O6 and O11, both of which were clinical isolates found in patients with nosocomial pneumonia [13]. The probability of *P. aeruginosa* PSU9449 being a human pathogen was calculated at 0.751, indicating its potential to cause disease in humans, as it matched 581 pathogenic families. Understanding its genetic characteristics is necessary for developing effective treatment strategies and infection control measures, particularly in hospital settings where it poses a significant threat to immunodeficient patients.

### 2.3. Functional Annotation

The functional annotation analysis by RAST showed that the subsystem features a coverage of 29% distributed in 387 subsystems (Table 2). The most frequently assigned subsystems were amino acids and derivatives = 479 (19.54%); carbohydrates = 258 (10.52%), and protein metabolism = 219 (8.93%). The RAST results were consistent with the top three subsystem distributions in the *P. aeruginosa* genome reported in previous studies [14,15]. Moreover, amino acids and their derivatives play a crucial role in protein synthesis as building blocks and are associated with the synthesis of various metabolic pathways, such as nucleotides. Moreover, these amino acids are related to survival under stress conditions and can contribute to virulence factor production, host interaction, and immune evasion [16]. Carbohydrates perform several essential functions in bacterial cells, beyond serving as energy sources and structural components. They are key constituents of many virulence factors, such as lipopolysaccharides (LPS), extracellular polysaccharides that form capsules, and biofilms. Carbohydrates are a major component of the extracellular polymeric substance (EPS) matrix, which embeds bacterial cell communities in biofilms. The primary carbohydrates involved in biofilm formation are alginate, Psl, and Pel [17]. In addition, protein metabolism is fundamentally important to survival, adaptability, stress response, enzyme or toxin production, and virulence factor development in *P. aeruginosa*, contributing significantly to its pathogenicity. Secreted enzymes such as protease, lipase and elastase can degrade host proteins, invade host cells, and produce toxins like Exotoxin A. The delivery of these enzymes or toxins involves protein secretion systems in *P. aeruginosa*, including five secretion systems (T1SS–T3SS, T5SS–T6SS). They not only facilitate the virulence factor but also enable some strains that produce beta-lactamases and aminoglycoside-modifying enzymes, which inactivate and modify antibiotics, leading to antibiotic resistance [18].

Interestingly, the categories of virulence, disease and defense were found in 63 RAST subsystems (2.57%) and phages, prophages, transposable elements, and plasmids were identified in 11 RAST subsystems (0.45%). These features were desirable aspects of a pathogenic strain.

### 2.4. Mobile Genetic Elements (MGEs)

In this study, a bioinformatic analysis of the draft genome predicted the presence of five MGEs in *P. aeruginosa* PSU9449, all of which were insertion sequences (ISs) carrying ARGs, as shown in Table 3. Plasmids were not detected. Node 3 contained three ISs, including IS*Pa6*, IS*Pa32*, and IS*Pa2*, which carried the *fosA* resistance gene. Node 1 harbored IS*Psy29*, which carried the *bla*_PAO_ and *aph (3’)-IIb* genes. However, no MGEs were identified in nodes 11 and 14, although they carried the *bla*_OXA-50_ and *catB7* genes, respectively. Additionally, node 18 contained IS*Pa22* without a resistance gene.

The IS elements are associated with genome rearrangement and plasticity. These elements are surrounded by open reading frames that encode transposases and can insert short DNA fragments into another location within a chromosome. ISs often play a role in mobilization and may carry antibiotic resistance genes. They facilitate the acquisition of resistance genes and transmit via horizontal gene transfer among bacterial populations [19,20]. According to ISfinder, IS*Pa6* and IS*Pa2* belong to the ISNCY family and originate from *P. aeruginosa,* with IS*Pa2* containing the exotoxin A (*toxA*) gene [21,22]. IS*Pa32* also originates from *P. aeruginosa* while IS*Psy29* originates from *Pseudomonas syringae,* and both are part of the IS3 family [23,24]. The ISs predicted in this strain were similar to those of a previous study that showed the IS elements in *P. aeruginosa* isolated from patients in Southern Thailand [12].

### 2.5. Bacteriocin-Encoding Genes

**Figure 2:**
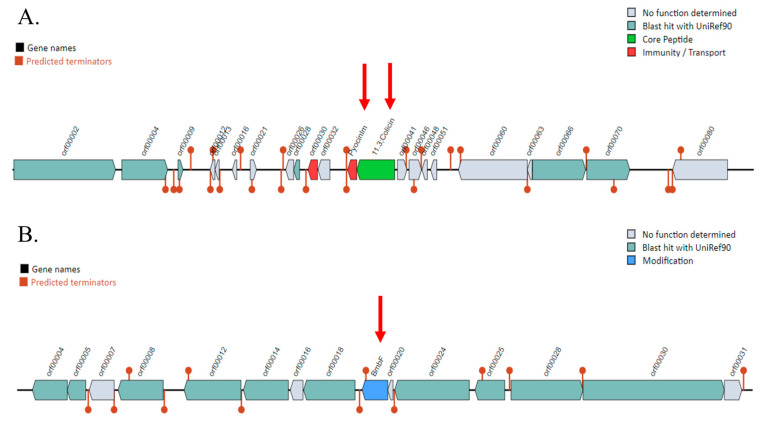
Prediction of bacteriocin gene clusters in the *Pseudomonas aeruginosa* PSU9449 genome using the BAGEL4 web server. The locations of the bacteriocin are indicated by red arrows. (**A**) Bacteriocin genes encoded on Contig 3, including pyocin classes such as pyocinIm and colicin. (**B**) The BmbF gene encoding bottromycin located on Contig 25.

The results of BAGEL4, used to predict bacteriocin-encoding genes, revealed two bacteriocin clusters identified in two Areas of Interest (AOIs). These clusters were in contig 3 (from 275,560 to 297,066 bp) and contig 25 (from 7667 to 27,667 bp) (Figure 2). Contig 3 encoded the pyocin-S2 immunity protein and colicin. Contig 25 contained the *bmbF* gene, which encoded a bacteriocin in the bottromycin class.

Pyocin, a bacteriocin produced by *P. aeruginosa*, has the advantage of being able to kill competing bacterial cells, including other strains of *P. aeruginosa* and other bacteria that share the same colonization niche. The soluble or S-type pyocin, which is colicin-like, is a protease-sensitive protein that exerts its killing effect by degrading chromosomal DNA through DNase activity [25,26]. The mechanism of pyocin S2 involves its entry into non-immune or sensitive *P. aeruginosa* cells under iron-limited conditions through a specific receptor, the siderophore pyoverdine receptor FpvAI (ferripyoverdine receptor type I) on the surface of *P. aeruginosa* cells. Additionally, an open reading frame (ORF) encodes an immunity protein (PyocinIm), which can attach to and inhibit the killing protein at the nuclease C-terminal domain, protecting the cell from the pyocin activity [27,28]. The properties of pyocin offer potential for developing new targeted antibacterial strategies, especially for combating antibiotic-resistant bacteria. For instance, a previous study demonstrated the potent activity of pyocin S2 against *P. aeruginosa* biofilms isolated from cystic fibrosis patients. This study showed that pyocin S2 effectively inhibits biofilm formation by *P. aeruginosa* and can prevent *P. aeruginosa* infections [29].

Bottromycins are macrocyclic peptides with strong antibacterial activity against Gram-positive bacteria, particularly MDR strains such as methicillin-resistant *Staphylococcus aureus* (MRSA) and vancomycin-resistant *Enterococci* (VRE). Bottromycins exert their antibacterial action by binding to the 50S ribosomal subunit and aminoacyl-tRNA binding site (A-site), thereby inhibiting protein synthesis [30].

### 2.6. CRISPR-Cas and R-M Sites

CRISPR (clustered regularly interspaced short palindromic repeats)-Cas (CRISPR-associated protein) systems offer a bacterial adaptive immune system to protect bacteria against foreign mobile genetic elements, which consists of a CRISPR array and Cas gene [31]. In *P. aeruginosa* PSU9449, we found five contigs with a CRISPR array, including 1, 24 (with cas6 type IF), 2, 8, and 9. The result is illustrated in Table 4.

CRISPR-Cas can help *P. aeruginosa* defend against invading genetic elements, such as a bacteriophage, prophage, or plasmid, as spacers in the CRISPR loci that recognize and destroy these elements upon re-invasion. This system can also control horizontal gene transfer, which involves the acquisition of MGEs carrying ARGs or virulence genes. Additionally, it provides evidence for the evolution of infection and can be used for genome editing and engineering novel therapeutics in *P. aeruginosa* [32,33]. However, R-M sites were not found in this strain.

### 2.7. Antibiotic Resistance Genes and Virulence Factors Profiling

Genomic analysis identified 51 ARGs with mechanisms including antibiotic inactivation, efflux pumps, antibiotic target alteration, and reduced antibiotic permeability. These genes collectively contribute to resistance against multiple antibiotic classes, including penicillin, macrolides, fluoroquinolones, aminoglycosides, carbapenems, and cephalosporins (Table 5). Based on genomic analysis, *P. aeruginosa* PSU9449 harbors multiple ARGs predicted to confer resistance to three or more antibiotic classes, suggesting its potential classification as an MDR strain, as it is predicted to be resistant to at least three or more antibiotic classes [34].

**Figure 3:**
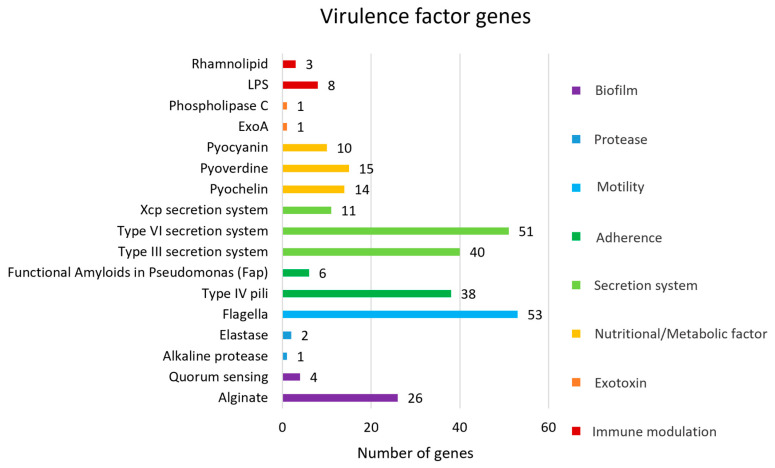
Virulence factor genes (VFGs) against virulence factor databases.

Bioinformatic analysis predicted 284 VFGs associated with biofilm formation, protease activity, motility, adherence, secretion systems, nutritional/metabolic factors, exotoxins, and immune modulation (Figure 3). The most prevalent VFGs were associated with flagella, which are crucial for motility, chemotaxis, surface colonization, adhesion to host tissues, and cell invasion [35]. The genes associated with flagellar expression and function, including *fli*, *che*, *fle*, *flg*, *flh*, and *mot* genes, play key roles in the assembly and formation of the basal body-hook complex and the regulation of the bacterial flagellum. Flagella are essential for bacterial motility, contributing to bacterial survival, host colonization, and pathogenicity [36]. The *alg* and *muc* genes are critical for the biosynthesis and regulation of alginate and mucopolysaccharides, which contribute to the biofilm matrix, bacterial biofilm formation, and virulence. Biofilm formation enhances bacterial survival and improves the bacteria’s ability to resist environmental stresses. The type 6 secretion system (T6SS) plays an important role in interbacterial competition (*hcp* gene) and interaction with host cells (*vgrG*, *pldA* gene). The T6SS is composed of several proteins that assemble into a structure resembling an inverted phage tail, which can puncture target cell membranes to deliver effector proteins. Additionally, the regulation of T6SS serves multiple virulence functions, including quorum sensing (QS) (*las*, *rhl* gene) and biofilm formation (*tssA* gene) [37]. Moreover, the type 3 secretion system (T3SS) plays an essential role in pathogenicity. It is a needle-like structure that can penetrate host cell membranes to deliver effector proteins into the host cell. These proteins are associated with facilitating disruption of host cell signaling (*exoS* gene), cytotoxicity (*exoU* gene), immune evasion (*exoT* gene), and manipulation of the host cell cytoskeleton (*exoY* gene) [38].

### 2.8. Pan-Genome Analysis and Phylogenetic Tree

**Figure 4:**
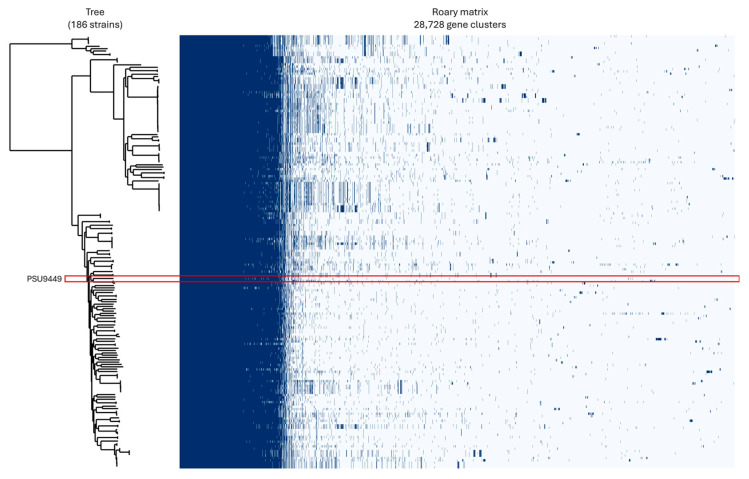
Pan-genome profiles of *Pseudomonas aeruginosa* PSU9449 (highlighted in red box) compared with 185 genomes from neighboring countries, illustrating genomic diversity.

The results of pan-genome analysis revealed a total number of 28,728 genes. These consisted of 3976 (13.84%) core genes, 908 (3.16%) soft core genes, 1640 (5.7%) shell genes and 22,204 (77.29%) cloud genes (Figure 4). The size of the accessory genome in this study, approximately 86%, is consistent with findings from other research, which report accessory genome sizes ranging approximately from 79% to 90% [12,39]. Generally, *P. aeruginosa* from various sources contains a wide array of accessory genes, many of which are acquired through horizontal gene transfer via plasmids, bacteriophages, transposons, or insertion sequences. These accessory genes are often associated with ARGs and VFGs [39,40,41]. This genomic plasticity underscores *P. aeruginosa*’s capacity to adapt to different environments and acquire traits that enhance its pathogenicity and resistance to treatment.

The sequence type (ST) of *P. aeruginosa* PSU9449 was determined through MLST, a method that analyzed seven housekeeping genes specific to *P. aeruginosa*. This analysis revealed that the strain belonged to ST3777, which represents a unique sequence type not previously reported in Thailand, as confirmed by data from PubMLST.

**Figure 5:**
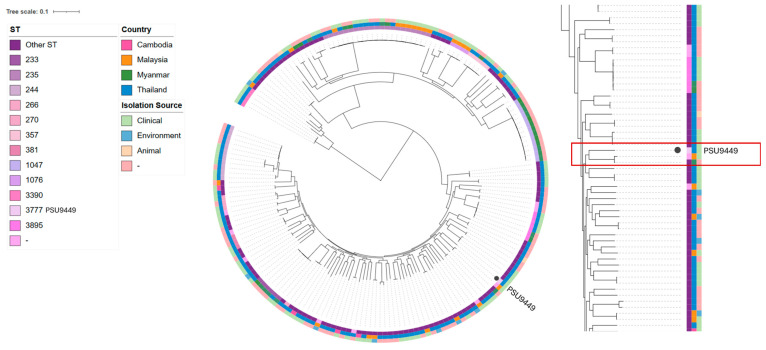
A single-nucleotide polymorphism (SNP) phylogenetic tree indicating the core genes of the *Pseudomonas aeruginosa* PSU9449 genome compared with 185 genomes in Cambodia, Malaysia, Myanmar, and Thailand. The innermost circles represent ST, country, and isolation source, respectively.

In Thailand, various STs are distributed differently, with ST1047 being predominant in Myanmar and ST-235 present in Malaysia, Myanmar, and Thailand. Our strain was closely related to *P. aeruginosa* HW001G from Malaysia and *P. aeruginosa* MyJU45 from Myanmar, forming a distinct clade (Figure 5). When compared with HW001G and MyJU45, our strain shared 5247 core genes. The majority of these shared genes are involved in essential biological processes such as metabolism, protein synthesis, energy production, and cell division. Notably, several shared genes are associated with virulence factors, including biofilm formation (e.g., *alg*, *muc*, and *las* genes), flagellar function (e.g., *flg*, *flh*, *fli*, and *mot* genes), and pyocyanin production (*phz* genes). Furthermore, these three strains share antimicrobial resistance genes, including *mexAB* and *fosA*, which are involved in antibiotic efflux and resistance to fosfomycin, respectively.

However, the unique genes of PSU9449, HW001G, and MyJU45 were 274, 364, and 715, respectively. Additionally, the interesting unique genes of PSU9449 include those related to Cas proteins (*cas6f*, *csy3*, *csy2*, *csy1*, *cas3*, and *cas1*), *lasR*, *bdlA*, *imm2* and *fliS.* The *lasR* gene is a key transcriptional activator in *P. aeruginosa* that plays a crucial role in the QS system, which is associated with the population density of the bacterial community by detecting and binding to autoinducer molecules [42]. The *bdlA* gene is a critical regulatory protein in the process of biofilm dispersion, which is the transition from a biofilm state to a free-living, planktonic state [43]. The *imm2* gene is a specific immunity protein of *P. aeruginosa* that protects it from its pyocin S2, as described above in relation to bacteriocin. Another virulence gene, the *fliS* gene, is involved in the assembly and function of the bacterial flagellum [36]. Therefore, all of these unique genes are related to the virulence factors of *P. aeruginosa* PSU9449.

## 3. Materials and Methods

### 3.1. Bacterial Strain and Antimicrobial Susceptibility Testing

*Pseudomonas aeruginosa* PSU9449 was isolated in 2017 from a throat swab of a hospitalized patient with underlying diseasesat Songklanagarind Hospital in Songkhla, Thailand. Initial species identification was performed using conventional biochemical tests according to Bergey’s Manual of Systematic Bacteriology, with confirmatory identification using Matrix-Assisted Laser Desorption/Ionization–Time of Flight (MALDI-TOF) mass spectrometry (Bruker Daltonik GmbH, Bremen, Germany). The isolate was cultured on Tryptic Soy Agar (TSA) media (HiMedia, Mumbai, India) and incubated at 37 °C overnight under aerobic conditions. After initial culturing, the strain was preserved in 20% glycerol at –80 °C for long-term storage. The antibiotic susceptibility of *P. aeruginosa* was tested against eight antimicrobial agents (amikacin, ciprofloxacin, imipenem, meropenem, ceftazidime, gentamicin, cefoperazone/sulbactam, and piperacilina/tazobactam) by the disk diffusion method. The results were evaluated following Clinical and Laboratory Standards Institute (CLSI) 2018 guidelines.

### 3.2. Genomic DNA Extraction and Whole-Genome Sequencing (WGS)

The GF-1 Bacterial DNA extraction kit (Vivantis, Subang Jaya, Malaysia) was employed for gDNA extraction following the manufacturer’s protocol. The purity and concentration of the gDNA extract were assessed using Thermo Scientific NanoDrop^TM^ 2000/2000c (Waltham, MA, USA) Spectrophotometers and confirmed through 1% *w*/*v* agarose gel electrophoresis. The purified gDNA was utilized for short-read WGS with 150 bp paired-end reads on the MGISEQ-2000 platform at the Beijing Genomics Institute (BGI, Shenzhen, China).

### 3.3. Genomic Assembly, Species Confirmation, and Annotation

Genomic reads were de novo assembled using Unicycler v0.5.0 [44], and quality assessment of genome assembly was evaluated using Quast V4.0 [45]. To perform species confirmation, the average nucleotide identity (ANI) was applied using FastANI v1.1.0 [46] compared with the *P. aeruginosa* PAO1 reference strain. Genome annotation was performed using Prokka v1.1.1 [47]. Functional annotations were assigned using Rapid Annotations using Subsystems Technology (RAST) (https://rast.nmpdr.org/rast.cgi (accessed on 30 October 2023)) [48,49,50] and EggNOG v5.0 [51] as well. The sequence types (STs) against traditional PubMLST typing schemes were identified in PubMLST (https://pubmlst.org/ (accessed on 30 October 2023)) [52].

### 3.4. Genomic Analysis

The *P. aeruginosa* serotypes were assigned using PAst 1.0 [53]. Identifying potential human pathogens was predicted using PathogenFinder 1.1 [54]. Bacteriocins were identified using the Bacteriocin Genome Mining Tool (BAGEL4) (http://bagel4.molgenrug.nl/ (accessed on 31 October 2023)) [55]. Mobile genetic elements (MGEs) related to ARGs and VFGs were identified using MobileElementFinder v1.0.3 [56]. CRISPR-Cas regions were investigated using the CRISPRCas Finder (https://crisprcas.i2bc.paris-saclay.fr/CrisprCasFinder/Index (accessed on 31 October 2023)) [57], and restriction-modification sites (R-M sites) were identified using Restriction-ModificationFinder 1.1 [58]. To identify ARGs, we employed ABRicate (https://github.com/tseemann/abricate (accessed on 3 November 2023)) and the Resistance Gene Identifier (RGI) (https://card.mcmaster.ca/analyze/rgi (accessed on 2 October 2023)) [59] against the Comprehensive Antibiotic Resistance Database (CARD) [60]. Virulence factors were detected using ABRicate (https://github.com/tseemann/abricate (accessed on 3 November 2023)) against the Virulence Factors Database (VFDB) [61] and VFanalyzer (http://www.mgc.ac.cn/cgi-bin/VFs/v5/main.cgi (accessed on 2 October 2023)). All bioinformatic tools were applied with default settings.

### 3.5. Genomic Diversity and Pangenome Insights Across Neighboring Countries

*P. aeruginosa* PSU9449 was compared with 185 genome assemblies of *P. aeruginosa* found in Thailand and neighboring countries (3 strains in Cambodia, 23 strains in Malaysia, 29 strains in Myanmar, and 129 strains in Thailand). These genomes were retrieved from the GenBank database [accessed on 31 October 2023]. The pan-genome profile was analyzed using Roary v3.13.0 with the default settings [62].

### 3.6. Single Nucleotide Polymorphism (SNP) Phylogenetic Tree Analysis

SNP-sites v2.4.1 was used for SNPs calling from core gene alignments [63]. A circular SNP tree based on core genes was generated by FastTree v2.1 with the default settings [64,65] and visualized using the Interactive Tree of Life (iTOL) v5 [66].

## 4. Conclusions

In summary, the comprehensive genomic analysis of *P. aeruginosa* PSU9449, a clinical colonizing isolate from Thailand, revealed important genetic features that contribute to its multidrug resistance and virulence potential. The strain, assigned to the previously unreported sequence type ST3777, carried several antimicrobial resistance genes (*fosA*, *aph (3’)-IIb*, *bla*_OXA-50_, and *catB7*) located within mobile genetic elements, along with virulence genes associated with flagellar function, the type VI secretion system (T6SS), and the type III secretion system (T3SS). Although phenotypic antimicrobial susceptibility testing showed susceptibility to all tested antibiotics, genotypic analysis identified resistance genes that may not be actively expressed under current conditions but could potentially confer resistance under selective pressure over time. These findings highlighted the isolate’s potential clinical relevance and underscored its significance within the broader context of regional antimicrobial resistance surveillance. A comparative genomic analysis further demonstrates that PSU9449 shared a close phylogenetic relationship with strains from Malaysia and Myanmar, by sharing some antimicrobial resistance genes (*mexAB* and *fosA* genes) and some virulence genes, which are involved in biofilm formation, flagella, and pyocyanin. These findings suggest possible cross-border dissemination and the emergence of regionally adapted lineages. The discovery of a novel sequence type and its genomic context highlights the need for continuous genomic monitoring to better understand the evolution, dissemination, and clinical impact of multidrug-resistant *P. aeruginosa* in Southeast Asia.

## Figures and Tables

**Table 1:** Genome Features of *Pseudomonas aeruginosa* PSU9449.

General Features	
Genome size (bp)	6,195,518
GC content (%)	66.4
Number of contigs	37
CDS	5607
Total RNA (tRNA + rRNA + tmRNA)	73 (68 + 4 + 1)
N50	399,445
L50	6
Number of ARGs	51
Number of VFGs	284

ARGs—Antimicrobial resistance genes, VFGs—Virulence factor genes.

**Table 2:** General features overview of subsystem information genes by RAST annotation.

Subsystem Features	Counts	%
Phages, prophages, transposable elements, plasmids	11	0.45
Cell wall and capsule	42	1.71
Cofactors, vitamins, prosthetic groups, pigments	196	7.99
Sulfur metabolism	39	1.59
Nitrogen metabolism	53	2.16
Metabolism of aromatic compounds	118	4.81
Fatty Acids, lipids, and isoprenoids	127	5.18
Iron acquisition and metabolism	51	2.08
Miscellaneous	45	1.84
Nucleosides and nucleotides	102	4.16
Amino acids and derivatives	479	19.54
Stress response	104	4.24
Dormancy and sporulation	2	0.08
Motility and chemotaxis	23	0.94
Respiration	114	4.65
Protein metabolism	219	8.93
Membrane transport	165	6.73
Phosphorus metabolism	42	1.71
Regulation and cell signaling	51	1.84
Cell division and cell cycle	0	0.00
Photosynthesis	0	0.00
DNA metabolism	77	3.14
RNA metabolism	57	2.32
Potassium metabolism	10	0.41
Virulence, disease, and defense	63	2.57
Secondary metabolism	4	0.16
Carbohydrates	258	10.52

**Table 3:** Mobile genetic elements carrying antibiotic resistance genes.

Contig	Position in Contig	Number of MGEs	Type of IS	ARGs	Phenotype
NODE 3	308,921–309,328	3	IS*Pa6*, IS*Pa32*, IS*Pa2*	*fosA*	fosfomycin
NODE 1	38,184–39,377	1	IS*Psy29*	*bla*_PAO_	ampicillin, amoxicillin, cefepime, ceftazidime
	25,022–25,828			*aph (3’)-IIb*	aminoglycoside
NODE 11	97,602–98,390	0		*bla*_OXA-50_	ampicillin, amoxicillin
NODE 14	119,289–119,927	0		*catB7*	chloramphenicol
NODE 18		1	IS*Pa22*		

**Table 4:** CRISPR (clustered regularly interspaced short palindromic repeats)-Cas (CRISPR-associated protein) systems in *P. aeruginosa* PSU9449.

Contig	Element	No. of Spacer/ Cas Gene (Cas Type)	Repeat Consensus/Cas Genes	Evidence Level
Node 1	CRISPR	12	GTTCACTGCCGTATAGGCAGCTAAGAAA	4
Node 24	CRISPR	9	GTTCACTGCCGTATAGGCAGCTAAGAAA	4
	Cas cluster	6 (IF)	cas1, cas3-cas2, cas6, csy1, csy2, csy3	
	CRISPR	12	TTTCTTAGCTGCCTACACGGCAGTGAAC	4
Node 2	CRISPR	1	GCGGCGGGTATCGGCGGATAACGCC	1
Node 8	CRISPR	1	TCATACCTTGCCCTCCAGTTCTTTGGCC	1
Node 9	CRISPR	1	GCCGACAACGGCAGCGAGCAGACCGT	1

**Table 5:** Antibiotic resistance genes against the CARD database.

Predicted Antibiotic Resistance Genes	AMR Gene Family
Antibiotic efflux	
*mexY*, *mexD*, *mexA*, *mexB*, *mexC*, *mexE*, *mexF*, *mexG*, *mexH*, *mexI*, *mexJ*, *mexK*, *mexL*, *mexV*, *mexW*, *mexM*, *mexN*, *mexP*, *mexQ*, *mexX*	Antibiotic efflux pump-type resistance-nodulation-cell division (RND)
*armR*	
*muxA*, *muxB*, *muxC*	
*opmB*, *ompD*, *opmH*, *ompJ*, *ompM*, *ompN*, *opmE*	
*parR*, *parS*	
*cpxR*	
*triA*, *triB*, *triC*	
*yajC*	
*rsmA*	
*pmpM*	Multidrug and toxic compound extrusion (MATE) transporter
*emrE*	Antibiotic efflux pump type small multidrug resistance (SMR)
*basS*	Pmr phosphoethanolamine transferase
*bcr-1*	Antibiotic efflux pump type major facilitator superfamily (MFS)
*cprS*, *cprR*	Pmr phosphoethanolamine transferase
Reduced permeability to the antibiotic	
*parR*, *parS*	Outer Membrane Porin (Opr)
Antibiotic inactivation	
*fosA*	Fosfomycin thiol transferase
*bla*_OXA-50_	OXA beta-lactamase
*aph(3’)-IIb*	APH(3’)
*pdc-374*	PDC beta-lactamase
*catB7*	chloramphenicol acetyltransferase (CAT)
Antibiotic target alteration	
*arnA*, *basS*, *cprR*, *cprS*	Pmr phosphoethanolamine transferase

## Data Availability

The genome of *P. aeruginosa* PSU9449 (MEDPSU_PAT_001) in this study has been deposited in the NCBI GenBank under BioProject number PRJNA1033800, with BioSample number SAMN38044900.

## References

[1] Santajit S., Indrawattana N. (2016). Mechanisms of Antimicrobial Resistance in ESKAPE Pathogens. Biomed. Res. Int..

[2] Ruekit S., Srijan A., Serichantalergs O., Margulieux K.R., Mc Gann P., Mills E.G., Stribling W.G., Pimsawat T., Kormanee R., Nakornchai S. (2022). Molecular characterization of multidrug-resistant ESKAPEE pathogens from clinical samples in Chonburi, Thailand (2017–2018). BMC Infect. Dis..

[3] Wareth G., Brangsch H., Nguyen N., Nguyen T., Pletz M., Neubauer H., Sprague L.D. (2024). WGS analysis of hypervirulent and MDR *Klebsiella pneumoniae* from Vietnam reveales an inverse relationship between resistome and virulome. Ger. J. Microbiol..

[4] Ngoi S.T., Chong C.W., Ponnampalavanar S.S.L.S., Tang S.N., Idris N., Abdul J.K., Gregory MJHusain T., Teh C.S.J. (2021). Genetic mechanisms and correlated risk factors of antimicrobial-resistant ESKAPEE pathogens isolated in a tertiary hospital in Malaysia. Antimicrob. Resist. Infect. Control.

[5] Nordmann P., Poirel L. (2019). Epidemiology and Diagnostics of Carbapenem Resistance in Gram-negative Bacteria. Clin. Infect. Dis..

[6] CDC (2019). Antibiotic Resistance Threats in the United States, 2019.

[7] Al-Orphaly M., Hadi H.A., Eltayeb F.K., Al-Hail H., Samuel B.G., Sultan A.A., Skariah S. (2021). Epidemiology of Multidrug-Resistant *Pseudomonas aeruginosa* in the Middle East and North Africa Region. Msphere.

[8] Masoud S., Njakoi G., Sholla S., Renatus D., Majigo M., Gangji R.R., Nyawale H., Mawazo A., Msafiri F., Ntukula A. (2024). Carbapenem resistance in *Pseudomonas aeruginosa* and *Acinetobacter baumannii* in Tanzania. Ger. J. Microbiol..

[9] Lee Y.-L., Ko W.-C., Hsueh P.-R. (2022). Geographic Patterns of Carbapenem-Resistant *Pseudomonas aeruginosa* in the Asia-Pacific Region: Results from the Antimicrobial Testing Leadership and Surveillance (ATLAS) Program, 2015–2019. Antimicrob. Agents Chemother..

[10] Zhao Y., Xie L., Wang C., Zhou Q., Jelsbak L. (2023). Comparative whole-genome analysis of China and global epidemic *Pseudomonas aeruginosa* high-risk clones. J. Glob. Antimicrob. Resist..

[11] Karlowsky J.A., Lob S.H., Siddiqui F., Akrich B., DeRyke C.A., Young K., Motyl M.R., Hawser S.P., Sahm D.F. (2023). In vitro activity of ceftolozane/tazobactam against multidrug-resistant *Pseudomonas aeruginosa* from patients in Western Europe: SMART 2017-2020. Int. J. Antimicrob. Agents.

[12] Chukamnerd A., Pomwised R., Chusri S., Singkhamanan K., Chumtong S., Jeenkeawpiam K., Sakunrang C., Saroeng K., Saengsuwan P., Wonglapsuwan M. (2023). Antimicrobial Susceptibility and Molecular Features of Colonizing Isolates of *Pseudomonas aeruginosa* and the Report of a Novel Sequence Type (ST) 3910 from Thailand. Antibiotics.

[13] Lu Q., Eggimann P., Luyt C.-E., Wolff M., Tamm M., François B., Mercier E., Garbino J., Laterre P.-F., Koch H. (2014). *Pseudomonas aeruginosa* serotypes in nosocomial pneumonia: Prevalence and clinical outcomes. Crit. Care.

[14] Rikame T., Borde M. (2022). Whole Genome, Functional Annotation and Comparative Genomics of Plant Growth-Promoting Bacteria *Pseudomonas aeruginosa* (NG61) with Potential Application in Agro-Industry. Curr. Microbiol..

[15] Valeeva L.R., Pudova D.S., Khabipova N.N., Shigapova L.H., Shagimardanova E.I., Rogov A.M., Tagirova T.R., Gimadeev Z.G., Sharipova M.R. (2023). The dataset on the draft whole-genome sequences of two *Pseudomonas aeruginosa* strains isolated from urine samples of patients with urinary tract diseases. Data Brief..

[16] Dai Z., Wu Z., Zhu W., Wu G. (2022). Amino Acids in Microbial Metabolism and Function. Adv. Exp. Med. Biol..

[17] Singh S., Almuhanna Y., Alshahrani M.Y., Lowman D., Rice P.J., Gell C., Ma Z., Graves B., Jackson D., Lee K. (2020). *Pseudomonas aeruginosa* biofilms display carbohydrate ligands for CD206 and CD209 that interfere with their receptor function. bioRxiv.

[18] de Sousa T., Hébraud M., Dapkevicius M., Maltez L., Pereira J.E., Capita R., Alonsa-Calleja C., Igarejas G., Poera P. (2021). Genomic and Metabolic Characteristics of the Pathogenicity in *Pseudomonas aeruginosa*. Int. J. Mol. Sci..

[19] Al-Nayyef H., Guyeux C., Petitjean M., Hocquet D., Bahi J. (2015). Relation between Insertion Sequences and Genome Rearrangements in *Pseudomonas aeruginosa* 2015. Bioinformatics and Biomedical Engineering (IWBBIO 2015).

[20] Galiot L., Monger X.C., Vincent A.T. (2023). Studying the Association between Antibiotic Resistance Genes and Insertion Sequences in Metagenomes: Challenges and Pitfalls. Antibiotics.

[21] Pritchard A.E., Vasil M.L. (1990). Possible insertion sequences in a mosaic genome organization upstream of the exotoxin A gene in *Pseudomonas aeruginosa*. J. Bacteriol..

[22] Sokol P.A., Luan M.Z., Storey D.G., Thirukkumaran P. (1994). Genetic rearrangement associated with in vivo mucoid conversion of *Pseudomonas aeruginosa* PAO is due to insertion elements. J. Bacteriol..

[23] Winsor G.L., Lo R., Sui S.J.H., Ung K.S.E., Huang S., Cheng D., Ching W.K.H., Hancock R.E.W., Brinkman F.S.L. (2005). *Pseudomonas aeruginosa* Genome Database and PseudoCAP: Facilitating community-based, continually updated, genome annotation. Nucleic Acids Res..

[24] Joardar V., Lindeberg M., Jackson Robert W., Selengut J., Dodson R., Brinkac L.M., Daugherty S.C., Deboy R., Durkin A.S., Giglio M.G. (2005). Whole-Genome Sequence Analysis of *Pseudomonas syringae* pv. phaseolicola 1448A Reveals Divergence among Pathovars in Genes Involved in Virulence and Transposition. J. Bacteriol..

[25] Denayer S., Matthijs S., Cornelis P. (2007). Pyocin S2 (Sa) Kills *Pseudomonas aeruginosa* Strains via the FpvA Type I Ferripyoverdine Receptor. J. Bacteriol..

[26] Sano Y., Matsui H., Kobayashi M., Kageyama M. (1993). Molecular structures and functions of pyocins S1 and S2 in *Pseudomonas aeruginosa*. J. Bacteriol..

[27] Michel-Briand Y., Baysse C. (2002). The pyocins of *Pseudomonas aeruginosa*. Biochimie.

[28] Elfarash A., Wei Q., Cornelis P. (2012). The soluble pyocins S2 and S4 from *Pseudomonas aeruginosa* bind to the same FpvAI receptor. Microbiologyopen.

[29] Smith K., Martin L., Rinaldi A., Rajendran R., Ramage G., Walker D. (2012). Activity of Pyocin S2 against *Pseudomonas aeruginosa* Biofilms. Antimicrob. Agents Chemother..

[30] Franz L., Kazmaier U., Truman A.W., Koehnke J. (2021). Bottromycins-biosynthesis, synthesis and activity. Nat. Product. Rep..

[31] Barrangou R., Horvath P. (2017). A decade of discovery: CRISPR functions and applications. Nat. Microbiol..

[32] Wheatley R.M., MacLean R.C. (2021). CRISPR-Cas systems restrict horizontal gene transfer in *Pseudomonas aeruginosa*. ISME J..

[33] Rath D., Amlinger L., Rath A., Lundgren M. (2015). The CRISPR-Cas immune system: Biology, mechanisms and applications. Biochimie.

[34] Magiorakos A.P., Srinivasan A., Carey R.B., Carmeli Y., Falagas M.E., Giske C.G., Harbarth S., Hindler J.F., Kahlmeter G., Olsson-Liljequist B. (2012). Multidrug-resistant, extensively drug-resistant and pandrug-resistant bacteria: An international expert proposal for interim standard definitions for acquired resistance. Clin. Microbiol. Infect..

[35] Feldman M., Bryan R., Rajan S., Scheffler L., Brunnert S., Tang H., Prince A. (1998). Role of Flagella in Pathogenesis of *Pseudomonas aeruginosa* Pulmonary Infection. Infect. Immun..

[36] Bouteiller M., Dupont C., Bourigault Y., Latour X., Barbey C., Konto-Ghiorghi Y., Merieau A. (2021). *Pseudomonas* Flagella: Generalities and Specificities. Int. J. Mol. Sci..

[37] Chen L., Zou Y., She P., Wu Y. (2015). Composition, function, and regulation of T6SS in *Pseudomonas aeruginosa*. Microbiol. Res..

[38] Horna G., Ruiz J. (2021). Type 3 secretion system of *Pseudomonas aeruginosa*. Microbiol. Res..

[39] Gómez-Martínez J., Rocha-Gracia R.D.C., Bello-López E., Cevallos M.A., Castañeda-Lucio M., Sáenz Y., Jimenez-Flores G., Cortes-Cortes G., Lopez-Garcia A., LoZano-Zarain P. (2023). Comparative Genomics of *Pseudomonas aeruginosa* Strains Isolated from Different Ecological Niches. Antibiotics.

[40] Subedi D., Vijay A.K., Kohli G.S., Rice S.A., Willcox M. (2018). Comparative genomics of clinical strains of *Pseudomonas aeruginosa* strains isolated from different geographic sites. Sci. Rep..

[41] Freschi L., Vincent A.T., Jeukens J., Emond-Rheault J.G., Kukavica-Ibrulj I., Dupont M.J., Charette S.J., Boyle B., Levesque R.C. (2019). The *Pseudomonas aeruginosa* Pan-Genome Provides New Insights on Its Population Structure, Horizontal Gene Transfer, and Pathogenicity. Genome. Biol. Evol..

[42] Kiratisin P., Tucker K.D., Passador L. (2002). LasR, a transcriptional activator of *Pseudomonas aeruginosa* virulence genes, functions as a multimer. J. Bacteriol..

[43] Morgan R., Kohn S., Hwang S.-H., Hassett Daniel J., Sauer K. (2006). BdlA, a Chemotaxis Regulator Essential for Biofilm Dispersion in *Pseudomonas aeruginosa*. J. Bacteriol..

[44] Wick R.R., Judd L.M., Gorrie C.L., Holt K.E. (2017). Unicycler: Resolving bacterial genome assemblies from short and long sequencing reads. PLOS Comput. Biol..

[45] Mikheenko A., Prjibelski A., Saveliev V., Antipov D., Gurevich A. (2018). Versatile genome assembly evaluation with QUAST-LG. Bioinformatics.

[46] Jain C., Rodriguez R.L., Phillippy A.M., Konstantinidis K.T., Aluru S. (2018). High throughput ANI analysis of 90K prokaryotic genomes reveals clear species boundaries. Nat. Commun..

[47] Seemann T. (2014). Prokka: Rapid prokaryotic genome annotation. Bioinformatics.

[48] Aziz R.K., Bartels D., Best A.A., DeJongh M., Disz T., Edwards R.A., Formsma K., Gerdes S., Glass E.M., Kubal M. (2008). The RAST Server: Rapid annotations using subsystems technology. BMC Genom..

[49] Overbeek R., Olson R., Pusch G.D., Olsen G.J., Davis J.J., Disz T., Edwards R.A., Gerdes S., Parrello B., Shukla M. (2014). The SEED and the Rapid Annotation of microbial genomes using Subsystems Technology (RAST). Nucleic Acids Res..

[50] Brettin T., Davis J.J., Disz T., Edwards R.A., Gerdes S., Olsen G.J., Olsen R., Overbeek R., Parrello B., Pusch G.D. (2015). RASTtk: A modular and extensible implementation of the RAST algorithm for building custom annotation pipelines and annotating batches of genomes. Sci. Rep..

[51] Huerta-Cepas J., Szklarczyk D., Heller D., Hernández-Plaza A., Forslund S.K., Cook H., Mende D.R., Letunic I., Rattei T., Jensen L.J. (2018). eggNOG 5.0: A hierarchical, functionally and phylogenetically annotated orthology resource based on 5090 organisms and 2502 viruses. Nucleic Acids Res..

[52] Jolley K.A., Bray J.E., Maiden M.C.J. (2018). Open-access bacterial population genomics: BIGSdb software, the PubMLST.org website and their applications. Wellcome Open Res..

[53] Thrane Sandra W., Taylor Véronique L., Lund O., Lam Joseph S., Jelsbak L. (2016). Application of Whole-Genome Sequencing Data for O-Specific Antigen Analysis and In Silico Serotyping of *Pseudomonas aeruginosa* Isolates. J. Clin. Microbiol..

[54] Cosentino S., Voldby Larsen M., Møller Aarestrup F., Lund O. (2013). PathogenFinder-Distinguishing Friend from Foe Using Bacterial Whole Genome Sequence Data. PLoS ONE.

[55] Van Heel A.J., de Jong A., Song C., Viel J.H., Kok J., Kuipers O.P. (2018). BAGEL4: A user-friendly web server to thoroughly mine RiPPs and bacteriocins. Nucleic Acids Res..

[56] Johansson M.H.K., Bortolaia V., Tansirichaiya S., Aarestrup F.M., Roberts A.P., Petersen T.N. (2020). Detection of mobile genetic elements associated with antibiotic resistance in *Salmonella enterica* using a newly developed web tool: MobileElementFinder. J. Antimicrob. Chemother..

[57] Couvin D., Bernheim A., Toffano-Nioche C., Touchon M., Michalik J., Néron B., Rocha E.P.C., Vergnaud G., Gautheret D., Porcel C. (2018). CRISPRCasFinder, an update of CRISRFinder, includes a portable version, enhanced performance and integrates search for Cas proteins. Nucleic Acids Res..

[58] Roer L., Hendriksen Rene S., Leekitcharoenphon P., Lukjancenko O., Kaas Rolf S., Hasman H., Aarestrup F.M. (2016). Is the Evolution of Salmonella enterica subsp. enterica Linked to Restriction-Modification Systems?. Msystems.

[59] Jia B., Raphenya A.R., Alcock B., Waglechner N., Guo P., Tsang K.K., Lago B.A., Dave B.M., Pereira S., Sharma A.N. (2017). CARD 2017: Expansion and model-centric curation of the comprehensive antibiotic resistance database. Nucleic Acids Res..

[60] Alcock B.P., Huynh W., Chalil R., Smith K.W., Raphenya A.R., Wlodarski M.A., Edalatmand A., Petkau A., Syed S.A., Tsang K.K. (2023). CARD 2023: Expanded curation, support for machine learning, and resistome prediction at the Comprehensive Antibiotic Resistance Database. Nucleic Acids Res..

[61] Chen L., Zheng D., Liu B., Yang J., Jin Q. (2016). VFDB 2016: Hierarchical and refined dataset for big data analysis—10 years on. Nucleic Acids Res..

[62] Page A.J., Cummins C.A., Hunt M., Wong V.K., Reuter S., Holden M.T.G., Fookes M., Falush D., Keane J.A., Parkhill J. (2015). Roary: Rapid large-scale prokaryote pan genome analysis. Bioinformatics.

[63] Page A.J., Taylor B., Delaney A.J., Soares J., Seemann T., Keane J.A., Harris S.R. (2016). SNP-sites: Rapid efficient extraction of SNPs from multi-FASTA alignments. Microb. Genom..

[64] Price M.N., Dehal P.S., Arkin A.P. (2009). FastTree: Computing Large Minimum Evolution Trees with Profiles instead of a Distance Matrix. Mol. Biol. Evol..

[65] Price M.N., Dehal P.S., Arkin A.P. (2010). FastTree 2—Approximately Maximum-Likelihood Trees for Large Alignments. PLoS ONE.

[66] Letunic I., Bork P. (2021). Interactive Tree Of Life (iTOL) v5: An online tool for phylogenetic tree display and annotation. Nucleic Acids Res..

